# Automatically Detect and Track Multiple Fish Swimming in Shallow Water with Frequent Occlusion

**DOI:** 10.1371/journal.pone.0106506

**Published:** 2014-09-10

**Authors:** Zhi-Ming Qian, Xi En Cheng, Yan Qiu Chen

**Affiliations:** 1 School of Computer Science, Fudan University, Shanghai, China; 2 Chuxiong Normal University, Chuxiong, China; Pacific Northwest National Laboratory, United States of America

## Abstract

Due to its universality, swarm behavior in nature attracts much attention of scientists from many fields. Fish schools are examples of biological communities that demonstrate swarm behavior. The detection and tracking of fish in a school are of important significance for the quantitative research on swarm behavior. However, different from other biological communities, there are three problems in the detection and tracking of fish school, that is, variable appearances, complex motion and frequent occlusion. To solve these problems, we propose an effective method of fish detection and tracking. In this method, first, the fish head region is positioned through extremum detection and ellipse fitting; second, The Kalman filtering and feature matching are used to track the target in complex motion; finally, according to the feature information obtained by the detection and tracking, the tracking problems caused by frequent occlusion are processed through trajectory linking. We apply this method to track swimming fish school of different densities. The experimental results show that the proposed method is both accurate and reliable.

## Introduction

There has been growing research interest in animal collective behavior due to its high scientific values and a wide range of potential applications [1]–[3]. From biological perspective, the study of swarm behavior in animals can provide us with a better understanding of how animals evolve. In computer science, there are techniques being used such as particle swarm optimization and ant colony optimization that use these social interactions to solve optimization problems. In engineering, the study of swarm behavior has been used to create groups of robots that are capable of interacting and working together.

Fish school is one of the most common biological swarms in nature. The schooling fish often swim in various shapes. This behavior is either spontaneous or for resisting attacks. But what is the principle behind the movement? How do fish schools benefit from these movements to survive? How could we get revelation of bionic algorithm from schooling (artificial fish swarm algorithm)? These problems have been intriguing many scientists, especially biologists, physicists and computer scientists. Since the 1970’s, in an attempt to answer these questions, there are already researchers from different fields, who have begun to research by quantitative analysis [4]–[10], but because of the variability of fish motion and the complexity of their environment, currently, the study of fish school behavior is still challenging.

The most informative way to study schooling behavior and to discover underlying principles is through acquiring and quantitatively analyzing the motion data of the fish school [11]–[16]. While manual analyses of collective motion is tedious, time-consuming and sometimes even impossible, video-tracking technology helps rapid and objective quantification of collective motion. As the rapid development of image acquisition devices and video tracking methods, it has become possible to measure the trajectory of each individual in a large group.

**Figure 1:**
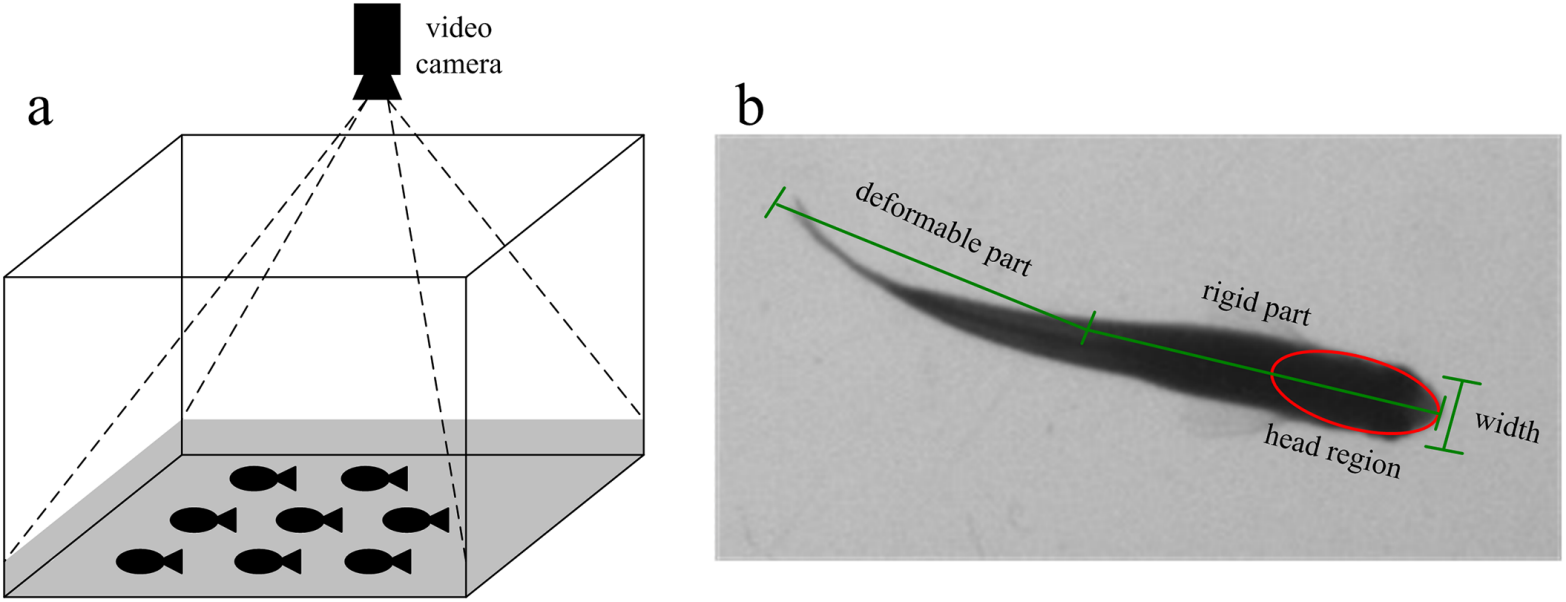
(a) Experiment environment; (b) Fish shape model.

When the schooling behavior is studied in a laboratory environment, a common experiment setup is to place a video camera vertically on top of a fish tank filled with shallow water as shown in Figure 1(a) for which the swimming motion can be approximately considered as a movement on a two-dimensional plane. While the problem of detection and tracking of fish school is related to the multi-target detection and tracking problem in the field of computer vision and pattern recognition, it has strong unique characteristics making it challenging and worth thorough investigation. In details, we are faced with the following two difficulties.

1. Detection problem: first, the shape of the fish is non rigid, the outline can’t be represented by one or several templates; second, the fishes’ texture information in the video image are less to detect and its location can’t be detected effectively only by texture features; finally, when the fish density is large, the targets will frequently occlude each other in the image. With the current target detection algorithm, it is difficult to detect the location of each target.
2. Tracking problem: first, the motion of fish swimming is so complex that the existing models cannot fully simulate; second, due to the higher degree of similarity among the fish, the use of a single feature method can hardly distinguish between different targets; finally, the detecting errors caused by fish occlusion will lead to a fragmentation in trajectory, adding more difficulties in tracking.

In order to overcome the above mentioned difficulties, we propose an effective method for tracking a large number of fish, which has the following advantages.

1. Based on the analysis of the fish shape, we propose a fish head region detection method that integrates local feature and geometric feature. First, the extremum detection of the entire image is implemented based on the gray distribution of the fish head region, and then according to the shape characteristics and contrast characteristics of the fish head region, the ellipse fitting and parameter estimation are conducted to the detected adjacent region of the extreme points, and the fish head region is further positioned accurately. This method comprehensively applies various features of the fish head appearance. It can accurately locate the fish head regions under different environments with fast computation and strong ability of anti-interference; second, the method simplifies the fish detection problem into the detection of one point and its adjacent region. Only by detecting partial shape information can we determine the fish’ position and better deal with the occlusion problem in fish school detection.
2. According to the detected target position, we first use Kalman filter to estimate its motion state, then apply feature matching method to realize data association. For targets failing to be predicted, we establish a compensation window to deal with that. Finally, in order to solve trajectory fragmentation caused by occlusion, we propose to use time and space information of trajectory fragments to complete the trajectory linking. The proposed tracking method takes full advantage of a variety of features and information in swimming fish school, able to deal with the complex motion of fish school and tracking problems caused by frequent occlusion effectively. Besides, with a low computational complexity, the method is more efficient in tracking targets in a large population.

Using the proposed method, we have successfully tracked the motion trajectories of fish schools of different densities. In order to measure the performance of the method, we compare the tracked results with the ground truth obtained by manual tracking. The result shows that the proposed method is accurate and robust.

### The Proposed Detection and Tracking Method

**Figure 2:**
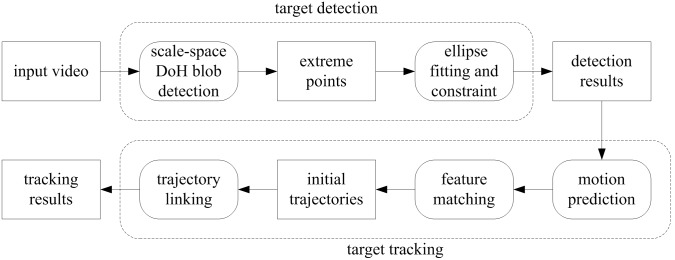
The diagram of the proposed method.

From Figure 1(b), we observe that the fish appears in a top view image as consisting of two parts: a rigid anterior part and a deformable posterior part that may swing to propel it. Since the fish head is rigid and its shape and grayscale distribution keep almost constant as the fish swims, an effective way to track fish in video is to find the fish head regions and associate them for each frame. Following this line of reasoning, the proposed method is designed to consist of several steps to track fish school. The first step is to detect the fish head from the video frames by using blob detection and ellipse fitting. The second step is to filter its motion state vector and predict its next value and utilize the predicted position together with feature matching results to associate the detected head regions of two consecutive frames. The final step is to deal with the possible fragmentation of the trajectories caused by occlusion via an effective trajectory linking method. An overview of the proposed method is shown in Figure 2. The following describes each step in detail.

### 2.1 Ethics statement

All experimental procedures were in compliance with the Institutional Animal Care and Use Committee (IACUC) of Shanghai Research Center for Model Organisms (Shanghai, China) with approval ID 2010-0010, and all efforts were made to minimize suffering. This study was approved by the Institutional Animal Care and Use Committee (IACUC), and written informed consent was obtained.

### 2.2 Target detection

For each frame image of the video sequence, we first detect the fish head regions. The detection method consists of two parts: scale-space DoH blob detection, ellipse fitting and constraint.

#### 2.2.1 Scale-space DoH blob detection

From Figure 1(b), we can see that the pixels inside the fish head region are considerably darker than the background ones, the head region is partially elliptical, and its width is greater than the rest part of the fish body. These characteristics show that fish head region appears like a blob and we first use blob detection to find the fish head region. In scale-space, it is an effective method to detect image blobs by using Determinant of Hessian (DoH) [17],[18], which reflects the local structural information of the image, and can better detect blobs of different scales in the image and well suppress the slender blobs in the image. The basic idea of scale space is: by introducing a scale parameter in image information processing model to obtain visual information at different scales through the continuous variation of the scale parameter; then, explore the substantive characteristics of image by combining all the information. The method of scale space absorbs the traditional single-scale visual information to the constantly changing dynamic analysis so as to obtain substantive characteristics of image more easily. It has been proved that scale space can be created by convolving the image with Gaussian kernel function [19]. The Gaussian kernel function has several good properties such as linearity, symmetry, separateness and so on, making it the kernel function best for the expression of scale space.

Suppose the pixel point is (*x*,*y*,*s*) in scale-space, where *x*, *y* are the point’s coordinates, *s* is the scale of the point, the Hessian matrix of the point is defined as:
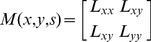
(1)where *L_xx_*, *L_yy_*, *L_xy_* are the convolution results of the Gaussian second order derivative and the point (*x*,*y*) at scale *s* respectively. The DoH of the matrix can be expressed as:



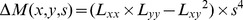
(2)Then the result in the blob detection is the extreme point of DoH responses for the position space and scale space:
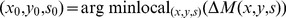
(3)


Since the head region gray value is less than the background region, we retain only the minimum extreme point. To improve the accuracy of the extreme point, we use tri-linear interpolation method to calculate the related parameters (coordinates and scale) of each extreme point. Tri-linear interpolation is a method of multivariate interpolation on a three-dimensional regular grid. It approximates the parameters of an extreme point within the local axial rectangular prism linearly, using data on the lattice points.

When scale-space DoH blob detection is carried out in the image, there always exists a stable extreme point at the center of the fish head region, which provides a reliable basis for the positioning of the head region. However, apart from the fish head regions, there may also exist extreme points in other regions. Therefore, it is necessary to pick up the extreme points that corresponding to the fish head regions out of all extreme points. In order to solve this problem, we fit ellipse for each extreme point according to the grayscale change of the extreme point region. If the extreme point is located in the center of the head region, the fitted ellipse can most reflect the characteristics of the head region.

#### 2.2.2 Ellipse fitting and constraint

There is a corresponding relationship between the Hessian matrix of the extreme point detected in the previous step and the second-order derivative matrix. The second-order derivative matrix can also be called the autocorrelation matrix, and its eigenvalues can represent the curvature of the orthogonal direction, and the change of curvature can reflect anisotropy degree of the regional structure. Based on this characteristic, we fit ellipse by using the second-order derivative matrix of the extreme point, estimate the grayscale variance of the extreme point region and finally find the location of the fish head.

Let the second-order derivative matrix (Hessian matrix) corresponding to the extreme point (*x*_0_, *y*_0_, *s*_0_) be *M*(*x*_0_, *y*_0_, *s*_0_). Then the eigenvalues and eigenvectors of the matrix can be expressed as:
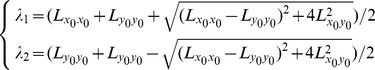
(4)

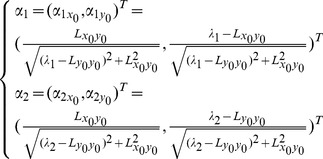
(5)where the eigenvectors *α*_1_, *α*_2_ correspond to the eigenvalues *λ*_1_ and *λ*_2_ respectively (|*λ*_1_|>|*λ*_2_|). The ratio of the eigenvalues is defined as 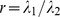.

**Figure 3:**
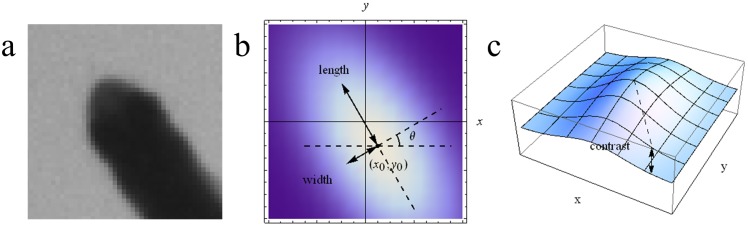
The illustration of the ellipse parameters. (a) The raw image of the fish head region; (b) (*x*_o_,*y*_o_) denotes the extreme point and the violet color shows the grayscale distribution of the extreme point region. Variables *length*, *width* and represent the long axis, short axis and angle of the fitted ellipse respectively; (c) The variable *contrast* in the direction of the z-axis represents the contrast change of the ellipse and its surrounding region.

Let the calculated extreme point (*x*_0_, *y*_0_) as the center of an ellipse; the ellipse major axis and the minor axis as the length and width of the fish head region, and the direction of the ellipse major axis as the direction of the fish head region. Then the length and width of the fish head region can be defined as 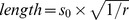, 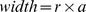; the orientation angle as 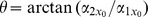, and the local contrast of each region as:
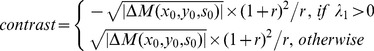
(6)Some parameters of the method are described in Figure 3.

The above method will obtain a plurality of candidate regions of head position. Because the fish head’s width is usually in a certain range, we first use the width constraint to remove the candidate regions generated by noise interference. Width threshold value *w* can be specified manually.

After the width constraint, there is still some false detection of candidate regions, which occur mainly in fishtail and fish body. In order to remove non-head regions from these candidate regions, we first perform image segmentation to identify fish regions from image. Image segmentation refers to the process of partitioning an image into a set of coherent regions. Since the image mainly contains two kinds of gray distribution (fish and background), thresholding method can distinguish them. Thresholding method is a common image segmentation method. It uses a threshold value to turn a grayscale image into a binary image. Here, we use Otsu method [20] to obtain fish regions from image. Otsu method is considered as a good thresholding method for image segmentation. It selects the threshold by minimizing the within-class variance of the two groups of pixels separated by the thresholding operator. Assuming the best segmentation threshold is *t*, *ω*_0_ and *ω*_1_ are the proportions of background pixels and foreground pixels in the image, *u*_0_ and *u*_1_ are the mean grays of background and foreground respectively, then the *t* value can be determined by the following equation:
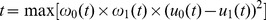
(7)


According to the results of segmentation, we use contrast constraint and angle constraint to remove non-head regions from the candidate regions.

Contrast constraint: The contrast of the fish head region and background is larger compared with the other parts. According to the results of equation (6), when 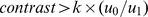, the region is considered to be an effective head region, where *k* is a contrast adjustment parameter. This constraint can effectively remove the candidate regions of the fishtail.

**Figure 4:**
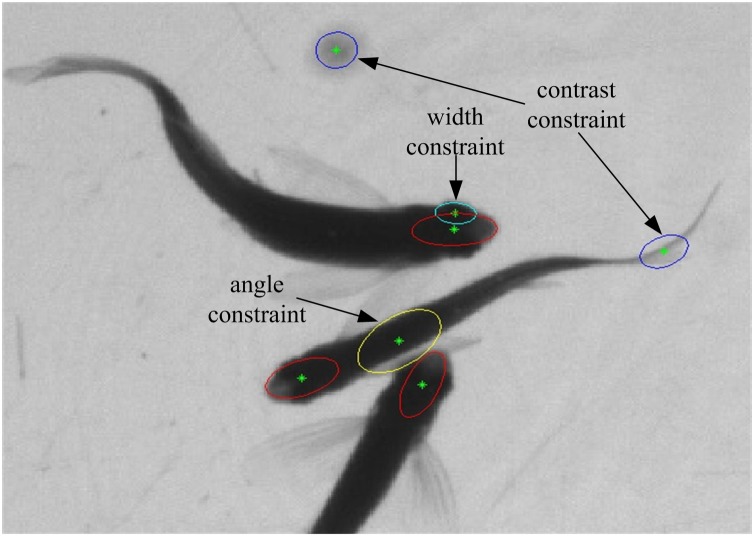
Candidate constraints based on width, contrast and angle.

Angle constraint: After the contrast constraint, if there are two or more candidate regions *cr*_1_, *cr*_2_…*cr_n_* in a segmented region, and their corresponding orientation angles and contrasts are *θ*_1_, *θ*_2_…*θ_n_* and *c*_1_, *c*_2_…*c_n_* respectively. If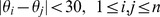, it is indicated that there is a phenomenon of duplicate detection. In this case, we reserve the candidate region of maximum contrast 
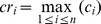
 and remove the other candidate regions. The reason for the angle setting is that when two fish are in mutual occlusion, angle between their head orientations is most likely greater than 30 degrees with few situations in which the angle is less than 30 degrees. We set that only when the angle is less than 30 degrees will it be constrained by the angle, ensuring that the occluded target will not be missed in most cases. For the small probability of missed detections, we will solve the problem by using trajectory linking method presented in section 2.3.3. Angle constraint can effectively remove the fish body candidate regions, ensuring that there is only one candidate region within a certain angle in a segmented region. Figure 4 shows an example of the candidate constraints.

### 2.3 Target tracking

After the detection of fish head region with the above method, we will track every detected targets in the whole video. The tracking method is generally described in three parts: motion prediction, feature matching and trajectory linking.

#### 2.3.1 Motion prediction

The fish’s motion state is represented by a four-dimensional state vector 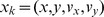, where *x* and *y* are the coordinates of the target center (ellipse center), *v_x_* and *v_y_* the speeds in the *x* direction and *y* direction. Define the observation variable *z_k_* = (*z_x_*, *z_y_*) to indicate the coordinates of the ellipse central after data association. Thus the targets’ motion state becomes able to be predicted by the Kalman filter [21]. Kalman filter is an optimal auto-regression data processing algorithm, which estimates a signal’ current value according to the previous estimated value and the most recent observational data, without all of the past observation data. The application of Kalman filter in tracking can transform a global search into local search to accelerate the tracking speed. In addition, when the target is blocked or interfered by other factors (the background noise or illumination change), Kalman filter’ predicted value can be used to replace the best associated target to improve the tracking performance. To simplify the model, assume target tracking system as a linear discrete system, then the system’ state equation and observation equation are described as:
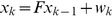
(8)

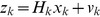
(9)where *F* and *H* are the target’ state transition matrix and observation matrix respectively, *w_k_* and *v_k_* the noises of state variable and observation variable respectively, both assumed to be independent and irrelevant zero-mean Gaussian noise. To estimate the motion state *x_k_* at *k*, we first predict the current state according to the previous estimated state. Because the fish motion between adjacent images generally differs slightly, hence the constant velocity model is applied to predict the state at the next time:



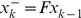
(10)

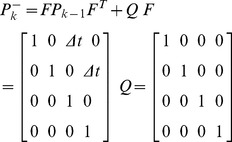
(11) where Δ*t* is the sampling time interval of two adjacent frames, 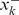 and 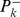 the model’s priori estimation of state variable and error covariance at k respectively. *Q* represents the covariance matrix of the state noise wk.

After data association, assume 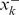
’s associated observation variable as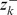, update the current state according to the equation below:
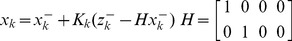
(12)

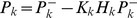
(13)Where 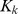 is the gain parameter at current time, which can be expressed as:
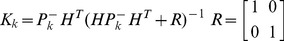
(14)where *R* is the covariance matrix of the observation noise *v_k_*.

**Figure 5:**
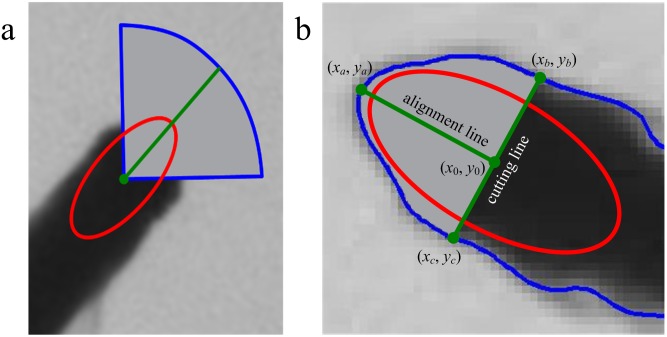
(a) The model of compensation window; (b) The segmentation model of matching region.

In the above motion prediction, the constant velocity model is chosen because of the similarities between fish school’ motion and the motion it describes, as well as its simple calculation for attainability. However, the motion of fish school in some cases is random, where constant velocity model cannot deal with. That is, the model itself cannot give a complete description of all the motions, bound to cause some errors in the subsequent tracking. Based on the statistical analysis of the test data, the change of fish head motion between two adjacent frames (1/30 second) is generally within ±45 degrees. Then we design a compensation window to track the targets failing to be predicted according to this law. As shown in Figure 5(a), the compensation window prescribes the detected ellipse’ direction as the target’s moving direction at current time, the target’ possible moving range as a quarter circle region with the ellipse center as the center and its long axis as the radius. If the prediction fails, the compensation window will be used for data association to make up for the shortage of the constant velocity model.

#### 2.3.2 Feature matching

Data association of state vectors and observations is a necessity for multi-target tracking. In order to optimize association accuracy, we employ feature matching. The key issue of feature matching is to find effective features that reflect the similarity among images of the same target and dissimilarity between images of different targets. The observation of sampling video finds that fish school generally moves horizontally in the shallow water, with very little vertical motion that can be ignored. That means the head region of the same target in different videos remains substantially constant. Based on the observed characteristics, we propose to use the width, area and grayscale information of fish head region for feature matching.

First, feature calculation. Based on the detection result in 2.2, active contour model [22] is used for the extraction of fish head contour and its initial region is the detected elliptical region. Assume (*x_i_*, *y_i_*) is a random point among the detected contour set, set the ellipse center (*x*_0_,*y*_0_) as the inner endpoint, search the range with a directional angle of *θ*±15 degrees for the farthest contour point (*x_a_*,*y_a_*) to the central point (*x*_0_,*y*_0_) as the outer endpoint. Then draw a line passing (*x*_0_,*y*_0_) and perpendicular to the line of inner and outer endpoints. Set the intersection points of the line and the contour as (*x_b_*,*y_b_*) and (*x*_c_,*y*_c_), then the line of these two points is the cutting line, the closed region enclosed by the cutting line and the contour is the target’ matching region, and the line of inner and outer endpoints and their directions is the alignment line of the matching region. Figure 5(b) shows the segmentation model of matching region.

Here we do not directly use the detected ellipse region for feature matching but the positional relationship between the ellipse center and head contour to redesign a segmentation method as matching region. This is because that the ellipse region is estimated according to the grayscale adjacent to the central point, subject to the change of light in the swimming of fish school. The proposed segmentation method takes advantage of the high stability of the ellipse center and the head contour, as well as active contour model’ high accuracy in contour description, which together contribute to the obtained matching region’ strong consistency between the adjacent images and therefore greatly improve the accuracy of feature matching.

After obtaining the matching region, the next is feature matching. Based on the degree of feature discrimination, we use cascade method for feature matching, which not only ensures the accuracy of matching, but also improves the matching speed.

Let the cutting line at *k*-1 as *L_k_*_-1_ and matching region *MR_k_*_-1_, then the width matching at *k* is expressed as:
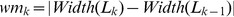
(15)where, *Width* is the width of the corresponding cutting line.

Area matching is expressed as:
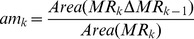
(16)where, △ is the symmetric difference set of the two matching regions after the alignment of alignment line, *Area* the area of the corresponding matching region.

Grayscale matching is expressed as:
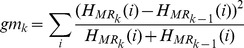
(17)where, *H* is the histogram of the corresponding matching region.

The final feature matching result is defined as follows:
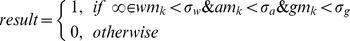
(18)


**Figure 6:**
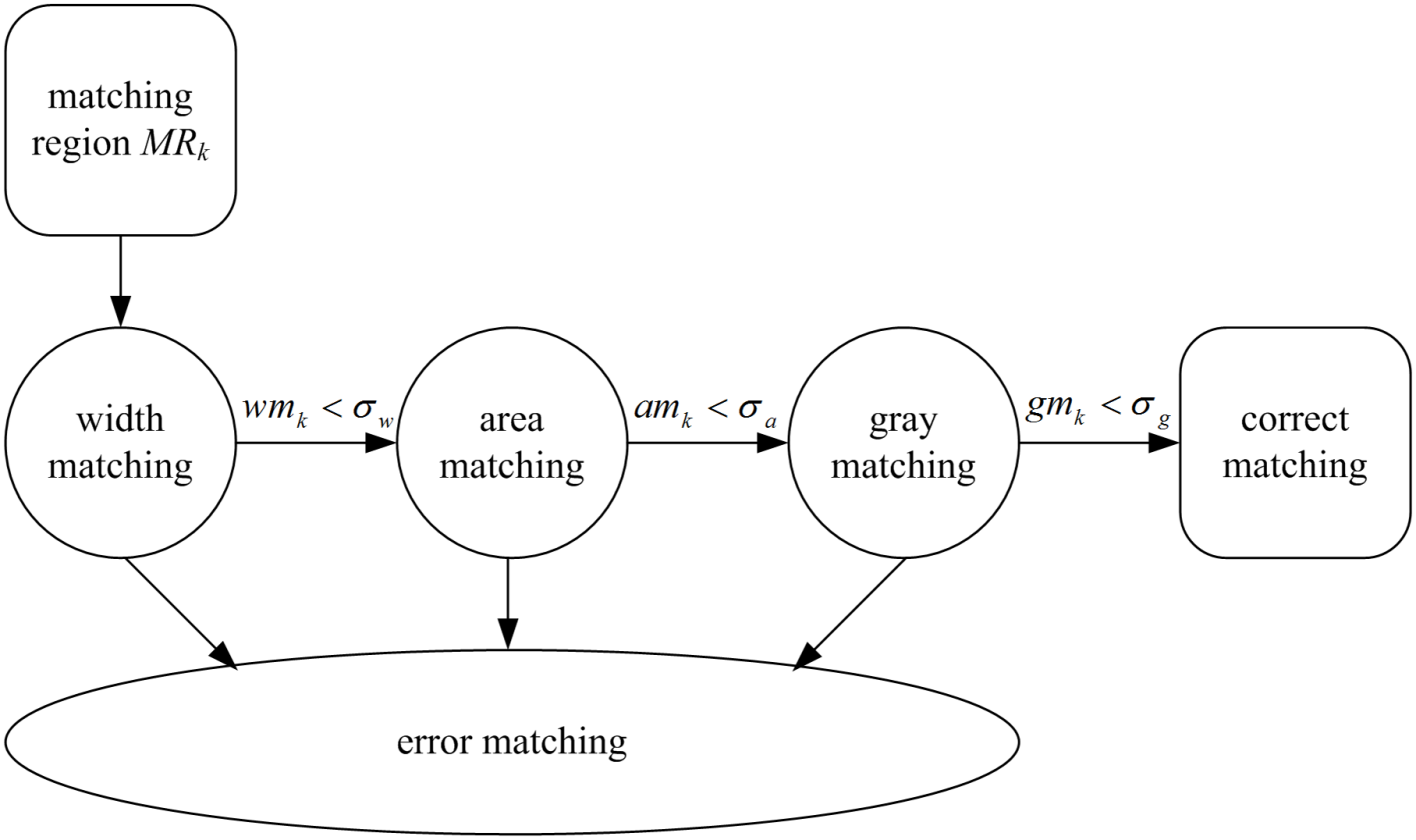
The process of feature matching.

Figure 6 shows the process of feature matching. If only one target’ matching region at *k* matches successfully, then the target is the associated observation variable. If more than one targets’ matching regions at *k* match successfully, the target with the smallest matching result 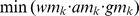 is the associated observation variable.

#### 2.3.3 Trajectory linking

The occlusion occurs frequently in swimming fish school, causing some detection errors and the failure of the complete matching between adjacent images and finally leading to the fragmentation of tracking trajectory. To solve this problem, we propose the following approach for trajectory handling on the basis of [23]:

1. If a state variable of the associated observation variable is found, update according to equation (12), and mark the state variable effective.
2. If no state variable of the associated observation variable is found, associate with a virtual observation variable, update according to 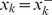 and mark the state variable ineffective. If no observation variable on the trajectory is associated in *T*_1_ consecutive frames, then the target probably keeps still, mark the trajectory incomplete and record the time *et* and position *ep* of the observation variable of the last effective state as the end tag of the trajectory.
3. If no observation variable of the associated state variable is found, we initialize the tracking and record the time *st* and position *sp* of the observation variable as the start tag of a new trajectory. The following tracking will see the two situations: A. if the observation variable is caused by error detection, it will last only a few frames of time, then we remove trajectory with an even less time of duration than this one; B. if the observation variable is generated by re-emerging target after occlusion, then mark the trajectory incomplete.

After the above process, we begin trajectory linking. Assume 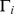 is an incomplete trajectory with end tag and 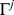 an incomplete trajectory with start tag. Define the constraint as below:
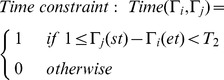
(19)


The above equation indicates that, if the initial time of trajectory 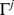 is later than the end time of trajectory 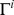, and time difference is less than *T*_2_, then the two trajectories meet time constraint.
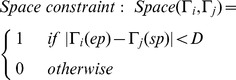
(20)


The above equation indicates that, if the distance between the end position of 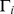 and start position of 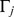 is less than *D*, then the two trajectories meet space constraint.

**Figure 7:**
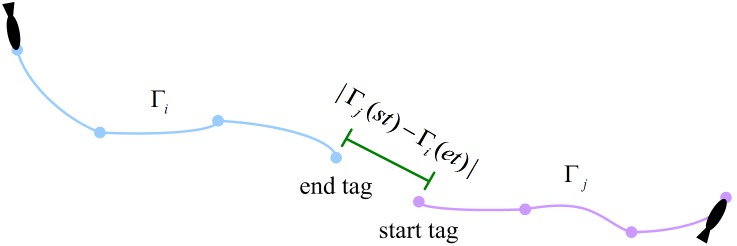
Trajectory linking based on time and distance.

If two trajectories meet both time constraint and space constraint at the same time, then they maybe belong to the trajectory fragments of one trajectory. Next, we conduct feature matching on the observation variables represented by the start tag and end tag. If the matching is successful, connect the two trajectories. Figure 7 shows an example of trajectory linking.

## Experiments and Discussions

We have conducted experiments to evaluate the performance of the proposed method in tracking multiple swimming fish. The experimental apparatus is shown in Figure 1(a). The fish are 2–3 cm long, and swim in a square acrylic tank of size 30 cm×30 cm filled with water of 3 cm deep. Fish behavior is relatively quiet and several motion modes (regular acceleration and deceleration, glide-and-burst, rapid and explosive motion) are present in the experimental data. A Flare 4M180-CL camera by IO Industries is placed above the tank at a distance to capture the entire arena. In order to evaluate the proposed method more challengingly, we leave the noise at the bottom of fish tank and the disturbance of the suspended matter in the water with no special processing. The computing facility includes a desktop computer with Intel I5 2.3 GHz processor, 4G RAM, GF9400 graphics card and Matlab programming environment. In order to test the tracking performance of different fish schools, we choose zebrafish (*Danio rerio*) with different densities in 3 groups: A1 (10 fish), A2 (20 fish), A3 (40 fish), the video for each fish group contains 500 frames. The time resolution of the camera is 30 frames per second and image resolution 2048×2040 pixels. The parameter settings are shown in Table 1.

**Table 1:** Parameter settings in the test process.

Detection Parameter		Tracking Parameter			Linking Parameter		
*w*	*k*	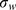	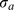	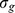	*T*_1_	*T*_2_	*D*
16–24	0.03	5	50	0.15	10	30	80

### 3.1 Evaluation of detection performance

We first carry out target detection for each frame image in the video. In order to quantify the performance of the proposed detection method, the precision and recall ratios that are widely adopted for evaluating object detection methods are used in the experiment. They are defined as follows:
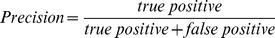
(21)

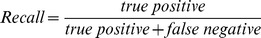
(22)where true positive is the total number of correctly detected regions in all frames; false negative is the total number of missed regions; false positive is the total number of wrongly detected regions.

In addition, in order to better evaluate the detection performance of the proposed method in the case of fish occlusion, we set up three additional evaluation criteria: *OR* (occlusion ratio), *ODR* (occlusion detection ratio) and *DT* (detection time).
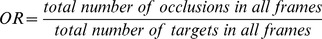
(23)

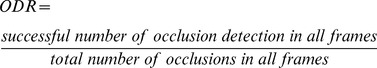
(24)

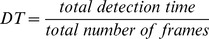
(25)


**Figure 8:**
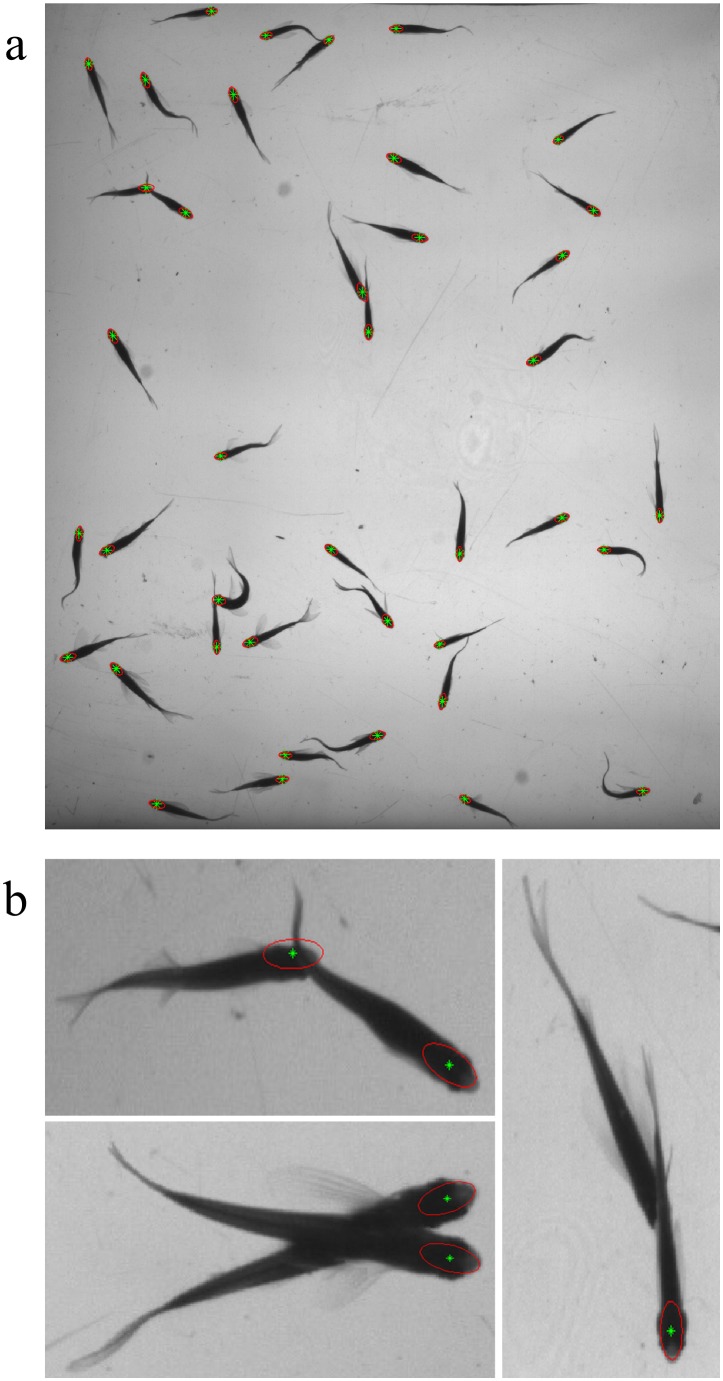
(a) Example of frame image illustrating the detection results of the fish head regions for a group of 40 fish; (b) Some examples of occlusion events efficiency resolved and a rare case where the detection failed.

The detection results are as shown in Table 2. From the results we can see that with the increase of fish school density, the occlusion ratio rises and the detection performance gradually declines. The fish school occlusion makes the head region invisible and then leads to the detection errors. In spite of this, the Precision ratio of the three groups of videos are maintained at over 0.971 and the Recall ratio are maintained at over 0.969, which fully proved the effectiveness of our detection method. Furthermore, occlusion detection ratio shows that, although fish occlusion brings some difficulties, the proposed detection method still demonstrates strong detection ability under occlusion. Because most occlusions are caused by fish body or tail rather than head, our method is then able to detect most occluded targets. Finally, seen from the detection time, the detection time in three groups are all within 1.9 seconds, and no significant change occurs with the increase of detection quantity, which indicates a good time performance in detecting target population. Figure 8 shows some detection results. As can be seen from Figure 8(b), when the target is occluded but the head region is visible, our detection method can detect the target’s location and direction according to the local information.

**Table 2:** Detection performance on different groups.

Group Size	Precision	Recall	Number of Occlusions	OR	ODR	DT
A1 (10 fish)	0.998	0.992	125	0.025	0.912	1.75
A2 (20 fish)	0.990	0.989	640	0.064	0.889	1.79
A3 (40 fish)	0.971	0.969	3040	0.152	0.846	1.88

### 3.2 Evaluation of tracking performance

After detecting and locating each fish, we then track them throughout the video to obtain their motion trajectories. To evaluate the proposed tracking method quantitatively, we associate the obtained trajectories 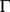 with ground truth trajectories *G* by using the approach proposed by [24]. Make 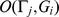 to indicate the frames where 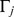 and 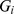 overlap, then the distance between two trajectories is defined as:
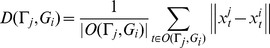
(26)where *x_t_* represents the target location on *t*. The above equation indicates the average distance between the obtained target position and ground truth target position over all frames. The cost of an association is defined as the sum of distances between the obtained trajectories and the associated ground truth trajectories. According to the cost, we can work out an optimal association A* that minimizes this cost.

Define two evaluation metrics to evaluate the tracking method. The first is *TCF* (trajectory completeness factor):
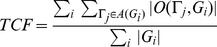
(27)where *A*(*G_i_*) is the set of obtained trajectories associated with *G_i_* in A*. It indicates the average ratio of one ground truth trajectory length covered by the obtained trajectories. The smaller the value is, the little the accuracy is. The second evaluation metric *TFF* (trajectory fragmentation factor) can be defined as:



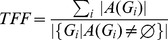
(28)It describes the average number of gained trajectories used to match one ground truth trajectory. Larger value means worse effect of the method in tracking the targets.

The tracking method consists of three parts: motion prediction, data association and trajectory linking. In order to better evaluate the proposed method, we use five methods with different schemes and compare them, and compared methods are shown in Table 3.

**Table 3:** Compared methods.

Number	Motion Prediction	Data Association	Trajectory Linking
Method1	None	Proposed	Proposed
Method2	Proposed	Nearest neighbor association [25]	Proposed
Method3	Proposed	Probabilistic data association [26]	Proposed
Method4	Proposed	Proposed	None
Method5	Proposed	Proposed	Proposed

**Figure 9:**
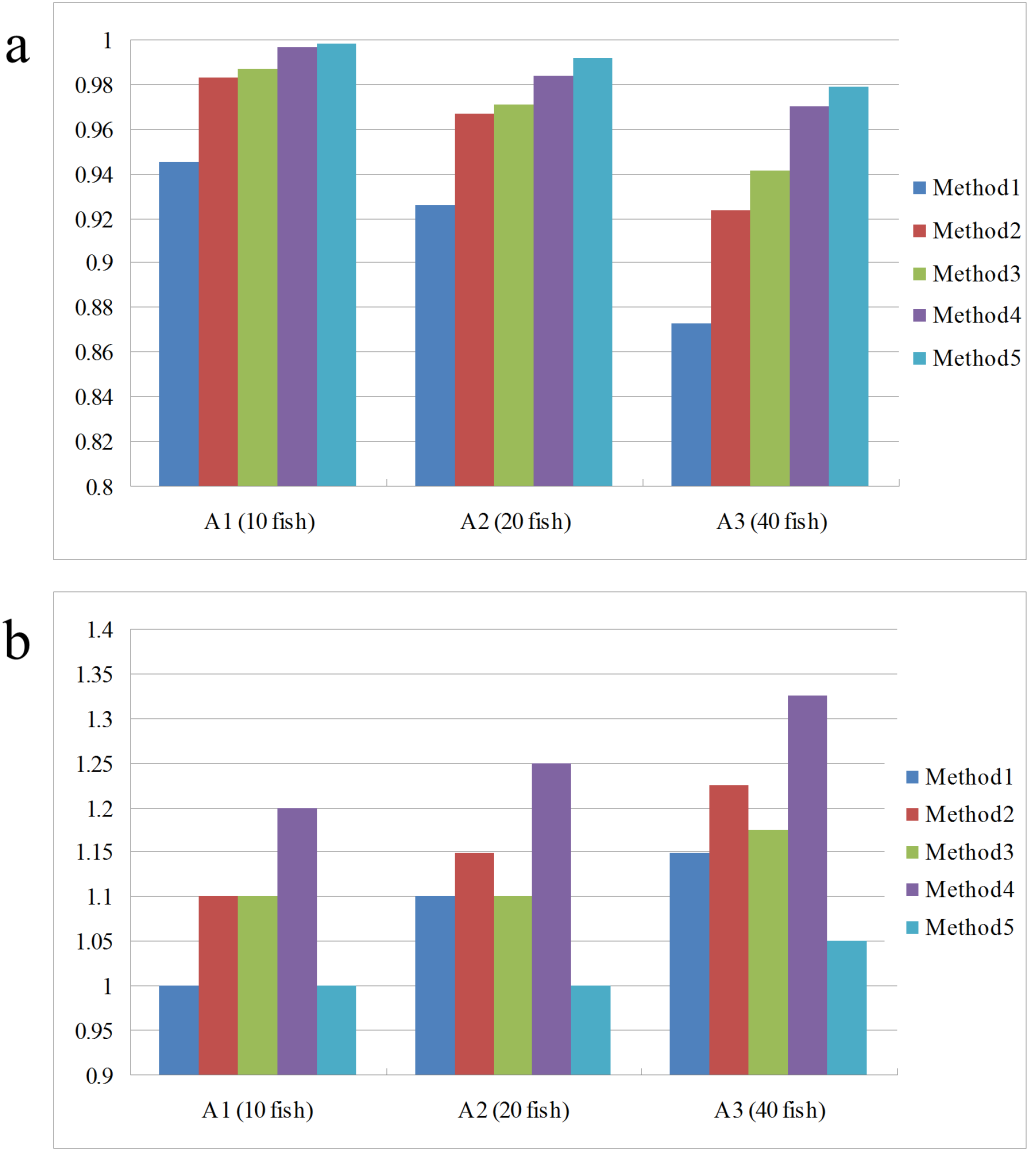
Performance of compared methods on two evaluation metrics. (a) TCF; (b) TFF. As fish density increases, tracking performance of all five methods falls. In comparison, the proposed method offers highest TCF values and lowest TFF values, indicating its performance is the best among the compared methods.

**Figure 10:**
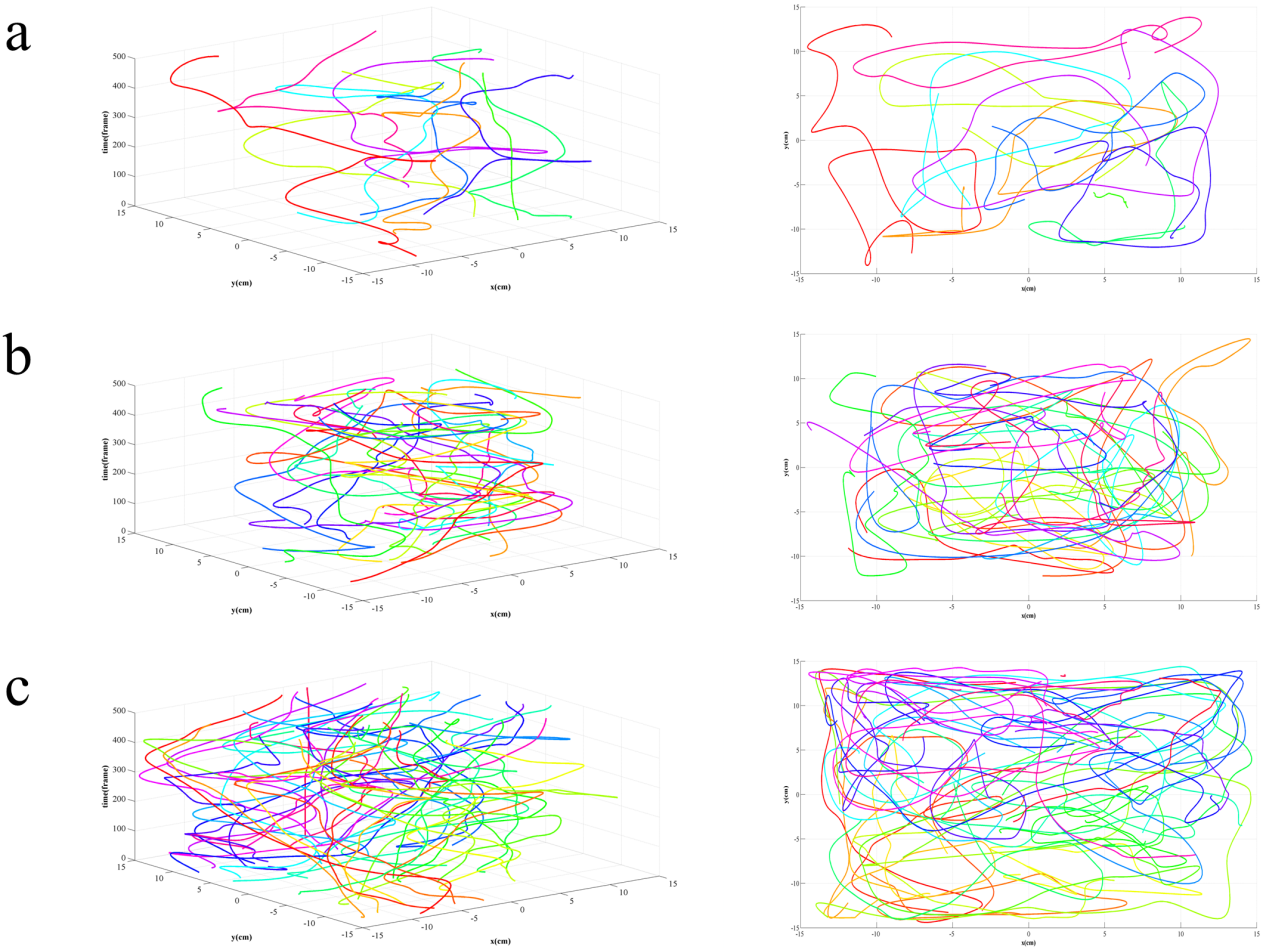
Tracking results on different groups with 16.7 seconds as duration. Left column: trajectory acquisition results with the time axis. Right column: trajectory acquisition results without the time axis. (a) A1 (10 fish); (b) A2 (20 fish); (c) A3 (40 fish).

Figure 9 shows the comparison results of different methods. Seen from the comparison of motion prediction, the adding of motion prediction performs much better than the single use of feature matching, especially in that, the more fish schools are, the more searching space for feature matching is needed, which will gradually lower the probability of successful matching and then lead to more tracking errors. By adding motion prediction into feature matching, the matching calculation drops and accuracy increases, thus tracking results are significantly improved. Seen from the comparison of data association, the tracking result of data association with feature matching performs significantly better than the nearest neighbor association [25] and the probabilistic data association [26], due to that the latter two only take account of fish school’ motion as the association basis. In actual tracking, the motion state of fish school is quite complex, with frequent occlusion. The motion information itself can hardly complete accurate association, while feature matching takes full advantage of the fish school's appearance information and keeps the consistency of targets in the complex motion. With the increase of fish schools, the tracking performance of all three methods declines because of more occlusion. However, the comparison finds that, the tracking performance of our tracking method declines much more slightly than the other two, showing that the proposed tracking method has strong robustness in multi-target tracking. Seen from the comparison of trajectory linking, the *TCF* and *TFF* using trajectory linking method is superior to the unconnected, which indicates that the gained trajectories becomes more intact after trajectory linking. In addition, with the increase of fish schools, the number of gained trajectories after trajectory linking also increases, with a more obvious effect in high-density population than low-density population, indicating that the proposed method can better deal with trajectory fragmentation problem caused by occlusion. Acquired trajectories using the proposed method in different groups are shown in Figure 10.

### 3.3 Discussions

In experimenting, we find out that for fish with rapid transition of swimming mode, Kalman filter will likely fail to predict a reasonable new state. Then we solve this problem by using a compensation window and trajectory linking method. In addition, we have performed preliminary experiments on images of golden shiner, paracherirodom innesi, tadpole and sperm, and results show that the proposed method can also detect and track the regions of their heads. The performance of the proposed method is closely related to the occlusion ratio. When the fish head region is occluded by other fish, the coordinate data of the target will be lost. The longer the occlusion time, the longer the coordinate data are lost. When the similarity between several matching regions is very high, feature matching may fail, which will lead to identity switch. The higher the density of fish group, the higher the probability of the occurrence of this situation. If the ratio between *BL*/*LA* (mean fish body length/length of arena) decreases, the occlusion ratio will go down, thus resulting in that fish can be tracked more easily. Conversely, if fish swim in polarized schools, it will cause severe occlusions, which will significantly increase tracking difficulties. The occlusion problem is the most difficult problem in multi-target tracking. Although we have tried to overcome it, the detection errors and tracking errors caused by occlusion cannot be completely avoided.

## Conclusion

This paper proposes an effective method for detecting and tracking multiple fish swimming in shallow water with frequent occlusion. Our contributions include a novel method for detecting multiple fish with possible occlusions based on robust image features around the head region. The method integrates the local extremum and ellipse fitting to locate the fish head region and better deal with the difficulties in fish school detection caused by factors such as variable appearances, frequent occlusion and small discrimination of texture region. Our second contribution is an effective method for first-pass tracking that combines Kalman filtering with feature matching, taking full advantage of the motion and appearance information of fish school to better cope with the tracking in complex motions. Our third contribution is a robust trajectory linking method as the second-pass of the tracking process in order to deal with frequent occlusion among fish. We have evaluated the proposed method on zebrafish schools of various densities in laboratory environment, and the results show its effectiveness and accuracy.

## Supporting Information

Movie S1 **Tracking result's demo video of 40 fish.**
(AVI)
